# A Hydrogen-Bromate Flow Battery as a Rechargeable Chemical Power Source

**DOI:** 10.3390/membranes12121228

**Published:** 2022-12-05

**Authors:** Natalia V. Kartashova, Dmitry V. Konev, Pavel A. Loktionov, Artem T. Glazkov, Olga A. Goncharova, Mikhail M. Petrov, Anatoly E. Antipov, Mikhail A. Vorotyntsev

**Affiliations:** 1 Faculty of Fundamental Physical and Chemical Engineering, Lomonosov Moscow State University, 119991 Moscow, Russia; 2 EMCPS Department, Mendeleev University of Chemical Technology of Russia, 125047 Moscow, Russia; 3 Federal Research Center of Problem of Chemical Physics and Medicinal Chemistry RAS, 142432 Chernogolovka, Russia; 4 Frumkin Institute of Physical Chemistry and Electrochemistry, Russian Academy of Sciences, 119071 Moscow, Russia

**Keywords:** crossover, flow battery, fuel cell, bromate anions reduction, bromide anions oxidation, redox mediator cycle

## Abstract

The hydrogen-bromate flow battery represents one of the promising variants for hybrid power sources. Its membrane-electrode assembly (MEA) combines a hydrogen gas diffusion anode and a porous flow-through cathode where bromate reduction takes place from its acidized aqueous solution: BrO_3_^−^ + 6 H^+^ + 6 e^−^ = Br^−^ + 3 H_2_O (*). The process of electric current generation occurs on the basis of the overall reaction: 3 H_2_ + BrO_3_^−^ = Br^−^ + 3 H_2_O (**), which has been studied in previous publications. Until this work, it has been unknown whether this device is able to function as a *rechargeable* power source. This means that the bromide anion, Br^−^, should be electrooxidized into the bromate anion, BrO_3_^−^, in the course of the charging stage *inside the same cell under strongly acidic conditions,* while until now this process has only been carried out in neutral or alkaline solutions with specially designed anode materials. In this study, we have demonstrated that processes (*) and (**) can be performed in a *cyclic* manner, i.e., as a series of charge and discharge stages with the use of MEA: H_2_, Freidenberg H23C8 Pt-C/GP-IEM 103/Sigracet 39AA, HBr + H_2_SO_4_; square cross-section of 4 cm^2^ surface area, under an alternating galvanostatic mode at a current density of 75 mA/cm^2^. The coulombic, voltaic and energy efficiencies of the flow battery under a cyclic regime, as well as the absorption spectra of the catholyte, were measured during its operation. The total amount of Br-containing compounds penetrating through the membrane into the anode space was also determined.

## 1. Introduction

Rechargeable power sources operating on the principle of redox flow batteries (RFBs) are an object of unrelenting interest for the scientific community, as they represent one of the principal ways to reduce the cost of each unit of electric energy, 1 kWh, of large-scale energy storage [1,2,3]. Among most well-known systems of this type one can point to those based on iron [4,5], vanadium [6,7,8,9,10], zinc-halide [11,12] and lithium-iodide redox-mediator RFBs [13], sodium-sulfur (Na-S) [14], hybrid lithium-TEMPO [15] and hydrogen-bromine RFBs [1,15,16,17]. A specific feature of the H_2_-Br_2_ system is the hydrogen reaction which requires a platinum catalyst at the anode [1,15,16,17], which is poisoned by transmembrane crossover of Br-containing species from the cathode space [18,19,20] where the electrode reaction takes place: Br_2_ + 2 e^−^ = 2 Br^−^.

The transition from the bromine- to bromate-based power sources [20,21,22] provides a chance to reduce this danger. Owing to the cation-exchange type of separating membranes in these devices, the transmembrane fluxes of both the reagent, the bromate anion (BrO_3_^−^), and the final product, the bromide anion (Br^−^), are significantly suppressed due to permselectivity effects [23,24,25]. At the same time, the concentration of a potentially hazardous Br-containing component in terms of its crossover, molecular bromine, Br_2_ (according to the study of K. Oh et. al. [18]), for which the membrane does not represent a permselective barrier, may be strongly diminished inside the catholyte. This occurs in the H_2_-Br_2_ system because this species is the *principal reagent* of the molar-range concentration, while in H_2_-BrO_3_^−^ systems, molecular bromine is merely an *intermediate* component in the course of the bromate—bromide conversion (autocatalytic redox-mediator catalysis by the Br_2_/Br^−^ couple, EC″ mechanism) so that its concentration can be maintained at a sufficiently low level via properly selected combination of the catholyte supply rate into the electrode space and the intensity of the current generated.

The principal reagent, the BrO_3_^−^ ion, is *non-electroactive* at the electrode surface so that its reduction proceeds via a *redox-mediator cycle*:

Br_2_ + 2 e^−^ = 2 Br^−^ at electrode surface; BrO_3_^−^ + 5 Br^−^ + 6 H^+^ = 3 Br_2_ + 3 H_2_O  in solution (1)

Global cathodic half-reaction: BrO_3_^−^ + 6 H^+^ + 6 e^−^ = Br^−^ + 3 H_2_O

Overall cell reaction: 3 H_2_ + BrO_3_^−^ = Br^−^ + 3 H_2_O

This means that the current passage is only ensured by the discharge of bromine molecules, while the generated bromide ions, Br^−^, react with bromate ions in the solution phase, thus restoring solute bromine molecules which may react again at the electrode. The set of reactions detailed in Equation (1) *might seem* to represent a particular case of the well-known EC’ mechanism [26,27,28,29,30,31,32,33,34,35,36,37,38,39,40,41,42,43], Equation (2):

Ox + n e^−^ = Red at electrode surface; A + Red = P + Ox  in solution(2)

where an electrochemically inert principal reagent, A, is transformed into its inert product(s), P, owing to a *catalytic redox-mediator cycle* based of an Ox/Red couple. It is well-established that its rate under steady-state transport-control regime is *proportional to the bulk-solution concentration of the catalytic species*, Ox^0^, while the transport rate of species A across the diffusion layer does not limit the process.

Theoretical analysis [44,45,46,47,48,49] of the bromate reduction process described by Equation (1) revealed its surprising features, which are *radically different from those for the cycle* in Equation (2). First, contrary to the conventional law for other reaction mechanisms, the highest rate of the process is ensured *not by an intensive convection*; quite oppositely, it is achieved for a *sufficiently thick diffusion layer*. Second, for a *very low* bulk-solution concentration of the catalytic species, Br_2_, the passing current can become *comparable* with, or even *exceed*, the *diffusion-limited one for the principal reagent,* BrO_3_^−^, which is well over 1 A/cm^2^ for its molar concentration range.

This difference in results for the bromate process, Equation (1), compared to those for the conventional EC’ mechanism, Equation (2), originates from the *autocatalytic* character of the former system. Namely, each passage of the redox cycle, Equation (1), transforms five Br^−^ ions into three Br_2_ molecules which (under suitable conditions) may give six Br^−^ ions at the electrode, i.e., the consumption of the principal reagent, BrO_3_^−^, leads to *an increase in the number of catalytic species*, Br_2_ and Br^−^, near the electrode surface. As a result, local concentrations of the latter species and, consequently, of the current may become so high that the process becomes limited *by the bromate transport into the reaction layer near the electrode surface*, which is proportional to the *bulk-solution concentration of bromate*.

These theoretical predictions were fully confirmed later by experimental studies of bromate reduction for various electrochemical configurations [50,51], including the most intriguing conclusion of a *drastic increase in the current for a less intensive convection*.

These fundamental studies paved the way towards power sources where bromate is used as an oxidant [21,22]. Very high values of the current and power densities (well over 1 A/cm^2^ and 1 W/cm^2^, respectively), as well as of the faradaic efficiency (over 93% in the course of a single catholyte passage through the cell), were attained for hydrogen-bromate MEA in the course of their discharges. They also revealed an excellent ability to increase the current and the power of such devices *proportionally to the membrane surface area*, from 0.5 cm^2^ to 50 cm^2^.

Even though the cathodic section of the H_2_-BrO_3_^−^ MEA was constructed and functioning in a close analogy to those for RFBs of other types the former was used as a *primary* power source based on the energy of the overall hydrogen-bromate reaction: 3 H_2_ + BrO_3_^−^ = Br^−^ + 3 H_2_O, i.e., *only within the discharge regime*. Until this study no attempts to perform this global process *in the opposite direction*, i.e., *to regenerate both bromate and hydrogen* have been made. At the same time, a hypothetic possibility for the H_2_-BrO_3_^−^ MEA to function in a *cyclic way*, i.e., as a set of discharge and charge stages, would strongly enhance the above merits of this device. The goal of this study was to shed light on this important aspect of bromate-based power sources.

The process of the oxidative transformation of a bromide solution, in particular up to the bromate one, has been extensively explored in the literature. Its first stage gives molecular bromine where the tribromide ion, Br_3_^−^, is present as an intermediate, in conformity with the thermodynamic analysis and experimental studies of the solution composition [52,53,54,55]. Even though thermodynamic analysis predicts a direct transition of Br_2_ to bromate in the course of a further oxidation process, its multi-electron character excludes it as a direct electrode reaction. Therefore, the subsequent formation of the oxygen-containing compounds of Br corresponding to its positive oxidation degrees proceeds via a set of chemical and electrochemical steps due to the generation of numerous intermediates. As a result, the rate and current efficiency of the further electrolysis may depend strongly on pH and on the electrochemical conditions.

Several publications have addressed the problem of the electrolytic production of bromates from bromides. The electrolysis of a bromide-containing disinfectant liquid (pH = 6–9) was studied to optimize its current output [56]. It was shown that the use of a RuO_2_/TiO_2_/Ti anode allows one to achieve a 67% current efficiency during the electrolysis of bromides at pH > 8.5 [57]. In addition, the electrolysis of bromides salts at the pH of the electrolyte (slightly alkaline) was carried out on a PbO_2_ anode with a current efficiency of 90% and a current density of 0.2 A/cm^2^ [58]. A current efficiency of 98–99% was achieved at pH 8.5–9.5 for the electrolysis of bromides on the RuO_2_/TiO_2_/Ti anode with the addition of Na_2_Cr_2_O_7_ [59]. Finally, a 100% current efficiency was reported for the electrolysis of bromide-containing solutions at a high pH on boron-doped diamond [60,61]

It should be noted that the main goal of these studies [56,57,58,59,60,61] was to optimize the current efficiency in order to minimize the effect of side reactions, rather than to minimize the applied voltage, while the latter is of primary importance for the energy-storing cycle. Besides this, the electrolysis was carried out an under alkaline pH, which is not compatible with the conditions of our study where the oxidative electrolysis has to be carried out for a *strongly acidized* bromide solution produced at the stage of bromate reduction inside the same discharge device.

The goal of this work has been to perform the transformation between bromate and bromide inside the hydrogen-bromate MEA *in a cyclic way*, i.e., to carry out the stages of charge and discharge *repeatedly* for the same overall reaction: 3 H_2_ + BrO_3_^−^ = Br^−^ + 3 H_2_O in both directions in order to assess the degree of the reagent and energy recovery as well as the prospects for its use for energy storage.

This study has been carried out for a model electrolyte (mixed sulfuric and hydrobromic acid solution) supplied into the electrolytic cell of the positive electrode of the hydrogen-bromate MEA: Freidenberg H23C8 Pt-C/GP-IEM 103/Sigracet 39AA, HBr + H_2_SO_4_ with a surface area of 4 cm^2^. The completeness of the bromide to bromate conversion and vice versa was monitored via measurements of the cell voltage and the cathodic polarization as well as the operando-spectroscopic control of the catholyte.

## 2. Materials and Methods

### 2.1. Design of a Hydrogen-Bromate Redox Flow Battery

**Figure 1:**
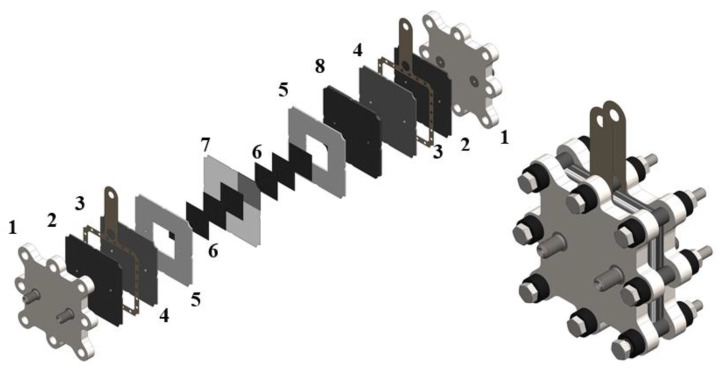
Scheme of hydrogen-bromate flow battery in the disassembled (centered) and assembled (bottom right) states: 1—end plates with fittings; 2—Viton gasket, 3—current collectors, 4—graphite foil, 5—electrode gaskets, 6—carbon paper electrode, 7—proton-exchange membrane with a pressed gas diffusion layer with a platinum catalyst (Pt/C loading 1 g/cm^2^), 8—glassy carbon plate (LLC El 6, Russia) with holes for electrolyte flow.

Studies of charge-discharge cycles were carried out on an MEA of the following structure: carbon paper Freudenberg H23C8 with a catalytic layer (Pt/C loading 1 mg/cm^2^) as the negative electrode, carbon paper Sigracet 39 AA (SGL Carbon, Wiesbaden, Germany) as the positive electrode, a proton exchange membrane GP-IEM 103 (Liaoning Grepalofu NewEnergy Co., Ltd., Panjin, China) for electrodes’ separation (geometrical surface area: 4 cm^2^). The structure of the cell of the hydrogen-bromate flow battery is represented in Figure 1.

The glassy carbon plates (LLC El 6, Moscow, Russia) used in the work only played the role of a current collector, contacting the porous carbon cathode by a flat surface. The supply/outflow of the catholyte into the carbon electrode was carried out through channels made in a Teflon flow frame on opposite sides of the square electrode space. Thus, the porous cathode functioned under the flow-through regime, without special distributing channels on the side of the glassy carbon plate.

Membrane samples were pretreated before testing by subsequent washing in tridistilled water under mild boiling, then treatment with a 3% hydrogen peroxide solution and storage in 2 M sulfuric acid.

Hydrogen was supplied to the negative electrode by a GH-25 generator (LLC “Metachrom”, Yoshkar-Ola, Russia). A water solution of 0.3 M HBr (Vekton, Moscow, Russia) + 3 M H_2_SO_4_ (Vekton, Voronezh, Russia) was circulated through the cathode space. Circulation was carried out using a Longerpump BT-100-1f peristaltic pump (Longer Precision Pump Co., Ltd., Hebei, China).

### 2.2. Methods

#### 2.2.1. Electrochemical Methods and Operando-Spectroscopic Control of the Catholyte Composition during Electrochemical Tests of the Hydrogen-Bromate Flow Battery

The charge—discharge characteristics of the hydrogen-bromate flow battery were measured using an Elins P-150X potentiostat-galvanostat (Electrochemical Instruments, Chernogolovka, Russia). Measurements of the cell voltage dependences on time were carried out in the galvanostatic mode: 0.3 A current for the electrooxidation of bromide ions (charging stage of the system), −0.3 A (±75 mA/cm^2^) current for the electroreduction of bromate ions (discharge stage of the system).

**Figure 2:**
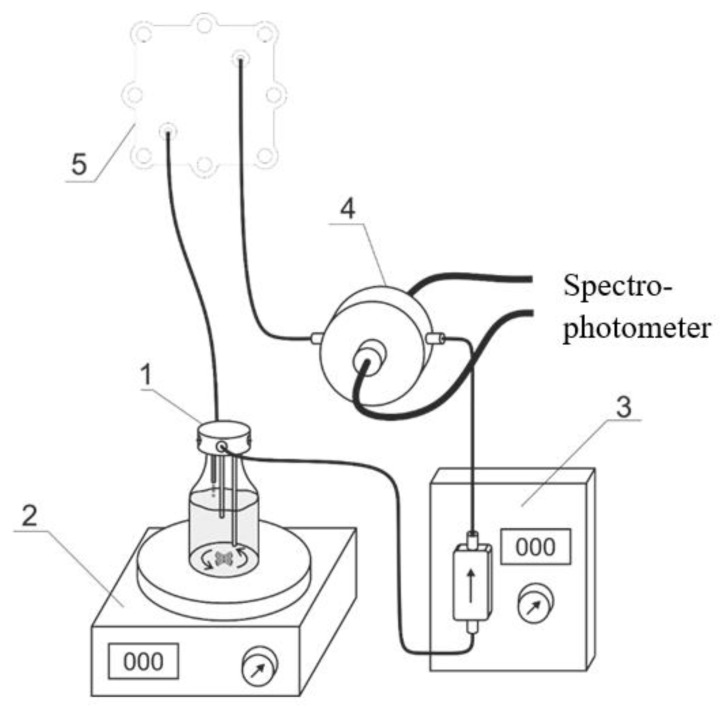
Experimental setup for studies of the hydrogen-bromate flow battery: tank with three connecting pipelines (1); magnetic stirrer (2); pumping unit (3); optical flow cell with fiber optic cables (4); hydrogen-bromate flow battery (5).

Optical absorption spectra were recorded intermittently during the charge—discharge tests of a hydrogen-bromate flow battery in order to control the evolution of the Br-containing electrolyte composition of the positive electrode. This was achieved with the use of Avantes StarLine AvaSpec2048 fiber optic spectrophotometer equipped with a cell of an original design indicated by the number 4 in Figure 2 [62]; interval between successive recordings: 20 s; optical path length: 290 μm. The optical flow cell was inserted into the electrolyte supply circuit of the positive electrode between the pump and the electrode. Thus, the measured spectra characterize the average electrolyte composition inside the reservoir. The spectrum of the optical flow cell with 3 M H_2_SO_4_ was recorded as a reference. The operation of the potentiostat and spectrophotometer were synchronized using a PC.

#### 2.2.2. Impedance Spectroscopy

To determine the cell resistance, hydrogen-bromate flow battery impedance hodographs were recorded in the frequency range of 1–50 kHz with an amplitude of 20 mV with the use of a P-45 potentiostat (Electrochemical Instruments, Russia). The high frequency cutoffs of the hodographs were used to determine the total ohmic resistance of the cell. The battery impedance hodograph was recorded by passing 3 M H_2_SO_4_ through the cathode and 0.5 L/h H_2_ through the anode.

#### 2.2.3. Cyclic Voltammetry for Assessing the Degradation of the Positive Electrode Material

Cyclic voltammetry was used to evaluate the degradation level of the carbon paper in the cell using a P-150X potentiostat-galvanostat (Electrochemical Instruments, Chernogolovka, Russia) connected in a two-electrode circuit. The positive terminal was connected to the positive electrode of the cell, where the bromate-containing electrolyte flows, while the negative terminal from the potentiostat was connected to the negative electrode, where a stoichiometric amount of hydrogen was supplied.

An analysis of the state of the carbon electrode after the charge/discharge tests of the hydrogen-bromate cell was carried out by the cyclic voltammetry method in sulfuric acid solution (3 M H_2_SO_4_), which was supplied to the cathode space instead of the bromate electrolyte. In this case, the hydrogen gas diffusion electrode was fed with hydrogen and acted simultaneously as both counter electrode and reference electrodes, relative to which the potential of the porous carbon electrode was cycled in the range between 0 and 0.8 V at a scan rate of 20 mV/s. Conclusions could be made regarding the cathode-material degradation on the basis of the observed change in the capacitive current related to the increase in the carbon fibers’ surface area.

#### 2.2.4. Investigation of a Hydrogen-Bromate Flow Battery Electrode Polarization

The electrode polarization during the operation of the hydrogen-bromate flow battery was measured using a reference electrode with a Luggin capillary of an original design [63] using a P-150X potentiostat-galvanostat (Electrochemical Instruments, Chernogolovka, Russia) in the voltmeter mode. The positive terminals from the potentiostat were attached to the electrode current collectors, the polarization of which was measured. The negative terminals were attached to the Ag/AgCl/KCl (saturated) silver chloride reference electrode, which had a potential on the scale of a standard hydrogen electrode of 0.198 V.

**Figure 3:**
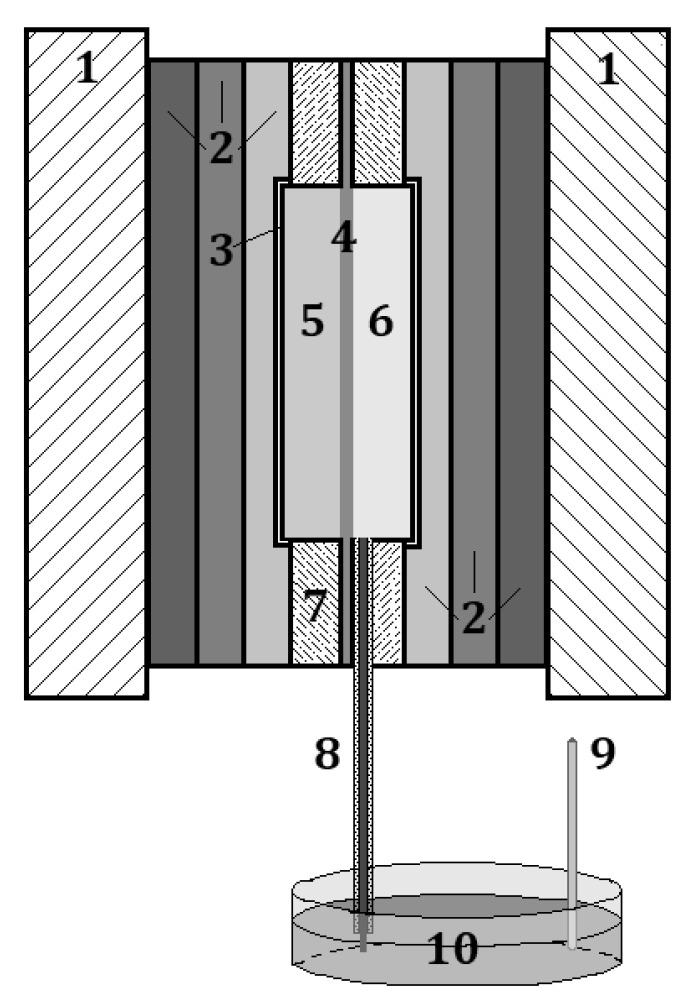
Connection scheme of the Luggin capillary and the reference electrode in the MEA. The attribution of numbers in the figure is described in Section 2.2.4.

The design of the MEA with the reference electrode is shown in Figure 3. It includes end plates (1), which provide mechanical contact by the ties with pins of the constituent parts of this design; current-collecting plates (2) and flow fields (3), which are designed to supply liquid reagents to the MEA. On Figure 3, the MEA consists of a proton-exchange membrane (4), electrode one (5), electrode two (6). The Luggin capillary device includes a gasket, i.e., a flow field limiter (7), and a Luggin capillary (8). The silver chloride electrode (9) is immersed into external electrolyte: sulfuric acid solution (10) is used as a reference electrode.

The silver chloride electrode was of a conventional construction: small reservoir filled by saturated KCl solution, into which silver wire coated with a layer of silver chloride was immersed. The reservoir possessed a liquid contact (through a frit made of porous sintered glass) with sulfuric acid solution, into which the end of the Luggin membrane capillary was also immersed, according to the diagram in Figure 3.

The Luggin thin-film capillary was a strip of the Nafion-211 proton-exchange membrane located between the membrane and one of the electrodes of the MEA (electrode one (5) in Figure 3 or electrode two (6) in Figure 3), and pressed to the membrane by a gasket-limiter of the flow fields for fixing the position of the capillary to the electrode. Thus, one end of the proton-exchange membrane strip is connected to the polarizable part of the electrode, and the other is placed in an additional electrolyte volume external to the MEA, in which there is an additional non-polarizable reversible electrode (reference electrode) with a constant and known value of electrode potential.

#### 2.2.5. Square-Wave Voltammetry for Evaluating the Crossover of Br-Containing Compounds

To evaluate the crossover of Br-containing compounds through the proton-exchange membrane, the square-wave voltammetry method was chosen due to its sensitivity for low concentrations of electroactive particles (instead of the standard cyclic voltammetry method, which is not sensitive enough for these estimates of bromide ion concentrations).

The concentration of HBr in the working electrode compartment of the three-electrode electrochemical cell was monitored by the electrochemical oxidation of the Br^−^ anion at the Pt working electrode. The electrode was a 1 mm stationary Pt disk, manufactured by soldering platinum wire into a glass tube. The end of this tube with a soldered Pt wire was flat polished. A Potentiostat Autolab 302N (“Metrohm”, Herisau, Switzerland) was used to control the three-electrode cell. Square potential pulses were applied of at height of 20 mV at 25 Hz frequency within a 0.5–1.3 V potential interval, with an average rate of potential change (equal to 5 mV/s) towards positive values of potential. Measurements were performed periodically. Prior to measurements, calibration was carried out by adding aliquots of HBr to the working electrode compartment of the cell.

## 3. Results and Discussion

The bromide oxidation process can be described as [57,64]:

6Br^−^ + 3H_2_O → BrO_3_^−^ + 5Br^−^ + 6H^+^ + 6e^−^, E^0^ = 1.41 V vs. SHE, (3)

This reaction was studied by carrying out a charging half-cycle by passing a current 75 mA/cm^2^ density through the MEA of the hydrogen-bromate flow battery for the initial electrolyte composition: 0.3 M HBr + 3 M H_2_SO_4_. When the cell voltage reached 1.9 V, the current direction was reversed, thereby switching to the discharge half-cycle.

**Figure 4:**
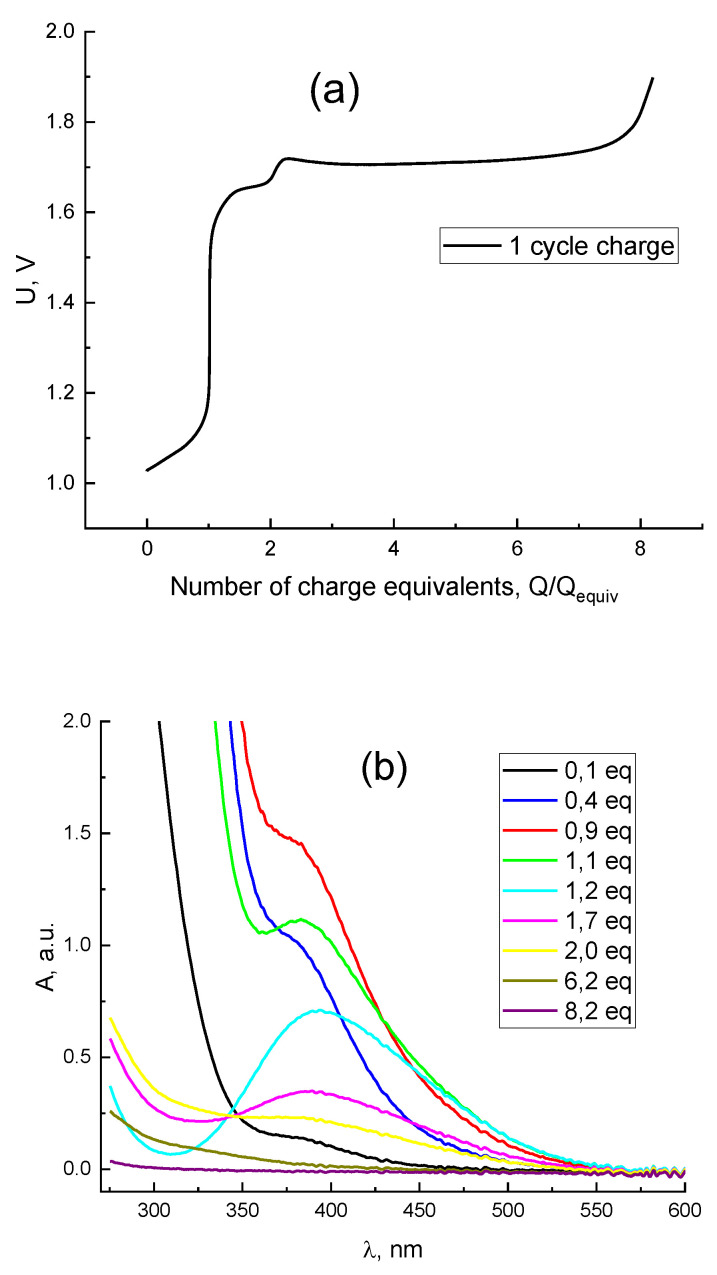
Charging curve of H_2_-BrO_3_^−^ flow battery (**a**) and the evolution of the corresponding spectrum of the catholyte (**b**) in the course of galvanostatic polarization by 0.075 A/cm^2^, starting composition of the positive-electrode solution: 0.3 M HBr + 3 M H_2_SO_4_, solution flow rate: 30 mL/min, exit of the negative electrode is open to the atmosphere.

Figure 4a demonstrates the resulting plot for the cell voltage vs. the number of equivalent charges passed during the process described above, i.e., the passed charge divided by the charge equivalent, Q/Q_equiv_. The latter is defined as the amount of charge required to change the oxidation degree of all Br atoms in the electrolyte by one unit which is equal to the product: Q_equiv_ = C × V × F where C and V are the initial concentration of bromide anions in the electrolyte and its volume, respectively, and F is the Faraday constant.

At the initial moments of charging with the transfer of 1 charge equivalent (1/6 from the theoretical charge for 13 mL 0.3 M HBr + 3 M H_2_SO_4_), part of the formed Br_2_ due to presence of Br^−^ transforms into Br_3_^−^ whose contribution dominates in the spectra of the solution due to its very large extinction. In this case, the side reaction of water oxidation proceeds simultaneously with the main oxidation reaction of bromide described in Equation (3), but to a lesser extent.

The concentration of bromide anion in the solution decreases after 1 charge equivalent flowing. Consequently, the quantity of tribromide anions drops down and a pure bromine peak appears on the UV-Vis spectra of the solution (Figure 4b). Then the Br_2_ concentration increases, confirmed by the spectral peak upsurge. Later, Br_2_ becomes oxidized and produces intermediate products with a positive oxidation state (+1), the HBrO signal appears on the Figure 4b, but its absorption peak goes beyond the measurement range of the UV—Vis method. The charging curve shows a strong voltage increase while passing 8 charge equivalents (Figure 4a). This indicates the exhaustion of electroactive Br-containing compounds in an oxidation state below (+5). Finally, we observed only the side reaction of water electrolysis with the O_2_ release after 8.2 charge equivalents.

**Figure 5:**
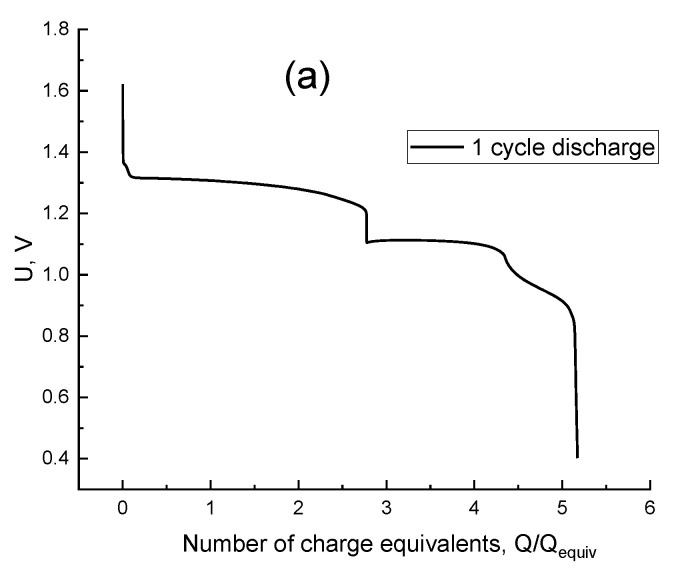

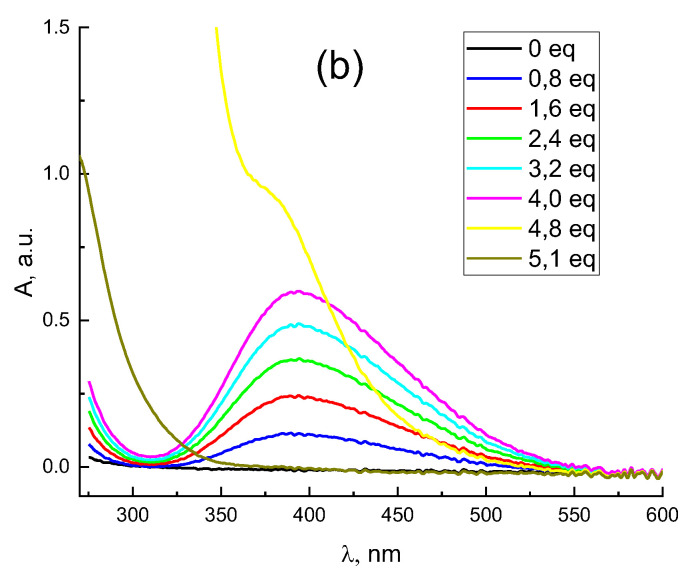
Discharging curve of a H_2_/BrO_3_^−^ flow battery (**a**) and evolution of the corresponding spectrum of the catholyte (**b**) in the course of galvanostatic polarization by −0.075 A/cm^2^, starting composition of the positive-electrode solution: product of stage (Figure 4a), reactant of the negative electrode: hydrogen, gas flow rate: 10 mL/min.

**Figure 6:**
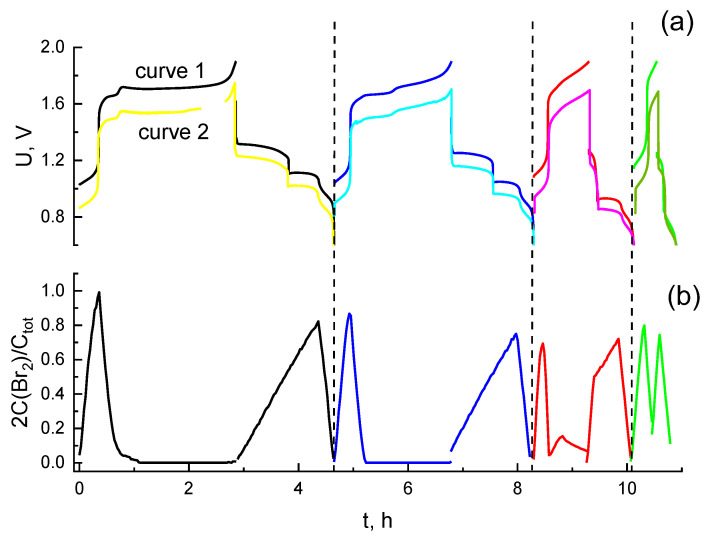
Charge—discharge (curve 1) and polarization (curve 2) curves of the positive electrode of a hydrogen-bromate flow battery (**a**), and a graph representing the variation in the *fraction* of Br atoms which are inside Br_2_ molecules at a time moment, t, which is equal to the ratio of the number of bromine atoms inside Br_2_ molecules to the initial content of bromine atoms in the system, i.e., 2 C(Br_2_)/C^0^(Br^−^) where C(i) is the molar concentration of species i; (**b**). Charging stage (Br^−^ oxidation): galvanostatic mode, current density: 0.075 A/cm^2^, initial composition of the catholyte supplied to the positive electrode: 0.3 M HBr + 3 M H_2_SO_4_, pumping rate: 30 mL/min, the negative electrode is open to the atmosphere. Discharge stage (BrO_3_^−^ reduction): galvanostatic mode, current density during discharge: −0.075 A/cm^2^, initial composition of the catholyte supplied to the positive electrode: solution at the end of the charge stage, hydrogen is supplied to the negative electrode at a rate of 10 mL/min.

**Figure 7:**
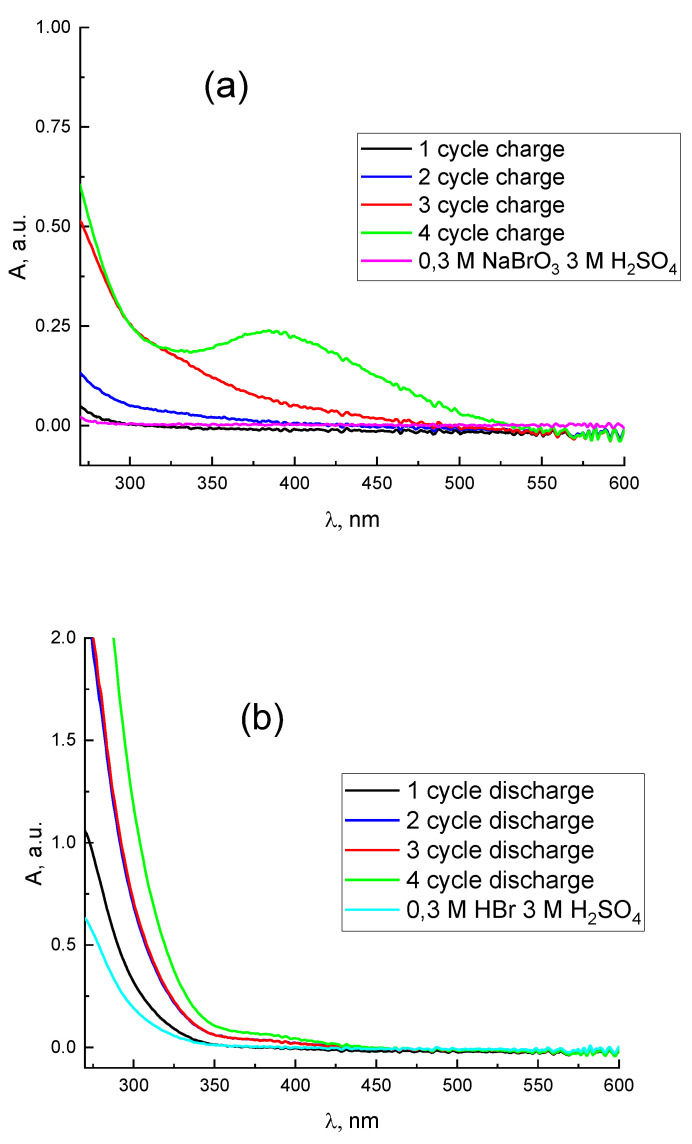
Spectra of the catholyte at the end of the charge (**a**) and discharge (**b**) of each cycle of operation of a hydrogen-bromate flow battery.

Before the discharge, the spectra indicate the absence of both Br_2_ and HBrO, which have absorption bands at 396 nm and 266 nm, respectively [65,66,67]. Therefore, the electrolyte contains only the bromate anion. The beginning of the discharge curve is demonstrated in Figure 5a; the hydrogen-bromate MEA cell voltage here corresponds to the process of converting bromate to bromine within the stage from its onset up to the passage of around 4.3 charge equivalents. The voltage drop within the subsequent stage of the discharge completion (from 4.3 charge equivalents to the end of the process) is caused by the absence of bromate anions in the catholyte. This stage corresponds to bromine reduction, in conformity with spectroscopy results (Figure 5b and Figure 6b). The diminution of the bromine concentration is accompanied by the growth of those of the bromide and Br_3_^−^ anions. Moreover, the concentration of the latter passes its maximum at the average oxidation degree of Br atoms in the system around +0.5, i.e., after the passage of around 4.8 charge equivalents, as one can observe from the spectroscopic data (yellow curve in Figure 5b which demonstrates the presence of the tribromide anion). After the completion of the discharge process the catholyte contains mostly bromide anions (Figure 7b). The additional absorption compared to the initial spectrum of 0.3 M HBr + 3 M H_2_SO_4_ (blue line in Figure 7b) is most likely the result of the presence of trace amounts of Br_3_^−^, because this complex gives an extremely intense UV absorption band [65,66,67]. Therefore, it can be concluded from Figure 4b and Figure 5b that the evolution of the electrolyte composition will be different during the charge and discharge processes of the hydrogen-bromate flow battery.

Figure 6 represents the results of a hydrogen bromate flow battery under cyclic testing with an extended duration over a voltage range of 0.4–1.9 V. In addition to the time dependence of the voltage (Figure 6a, curve 1), the potential of the positive electrode relative to the silver chloride reference electrode was registered (Figure 6a, curve 2). The processing of catholyte spectra in these tests was carried out according to the procedure described in the work of Petrov et al. [68] to isolate the molecular bromine fraction from the overall absorption spectra. The result is demonstrated in Figure 6b as a dependence of the relative amount of bromine atoms in the form of Br_2_ molecules with regards to time. While collating the curves for cycles 2–4 with the previously discussed data for the first cycle a noticeable difference was revealed: as the cycle number increases, its duration decreases, mainly due to the diminishing of the charging half-cycle stage, responsible for the rise of the bromine oxidation state from +1 to +5. As a result, the catholyte contains a bromate ion concentration decreasing from cycle to cycle by the moment the system reaches the current reversal voltage (1.9 V). At the same time, the proportion of bromine atoms in the form of HBrO increases. This corresponds to an increase in UV absorption (Figure 7a) compared to an acidic solution of sodium bromate, caused by intermediates with bromine oxidation states +1, +3 for cycles 2–3. A noticeable contribution of molecular bromine manifests in the fourth cycle. In this way, the average oxidation degree of bromine atoms in the catholyte by the end of the charge process decreases significantly from cycle to cycle: from ~ +5 after the first charge to less than +1 after the fourth charge.

Thereafter, the duration of the discharge stages also decreases. The plateau on the voltage-time curves which was attributed to the process of bromate reduction with the transformation into bromine in the description of the first cycle almost disappears for these cycles. The increase in the amounts of bromine intermediates in transitional oxidation states also affects the completeness of catholyte reduction by the end of the discharge process, as shown in Figure 7b, where the proportion of residual Br_3_^−^ is enhanced. On the other hand, the average oxidation degree of Br atoms at the end of the discharge stage was close to −1 in both the first and fourth cycles. The progressive loss of the passing charge in the course of the discharge state was significantly lower than that in the course of the charging stage.

Table 1 represents the calculation results for each of the four cycles, namely:

- Charges during the charging and discharging half-cycles, Q_charge_ and Q_discharge_, respectively;
- Average voltage during charging and discharging half-cycles, U_charge_ and U_discharge_ respectively;
- Coulombic efficiency (ηQ)—the ratio of the charge passed during discharge to the charge passed during charge in percent;
- Voltaic efficiency (ηU)—the ratio of the average discharge voltage to the average charge voltage in percent;
- Energy efficiency (ηE) of a hydrogen-bromate flow battery charge—discharge tests—the multiplication product of Coulombic and voltaic efficiencies;
- Capacity utilization (Q_discharge_/Q_tot_)—the ratio of the charge received while discharging the battery to the theoretical charge in percent (6 × F × c × V, where F is Faraday’s constant, c is the concentration of electroactive compounds, V is the electrolyte volume).

The results of capacity utilization (Q_charge_/Q_tot_) for cycles 1 and 2 are overestimated because of the contribution of the side reaction of water electrolysis. The battery characteristics are at a level high enough for practical use. The values of coulombic, voltaic and energy efficiencies are high, but the charge and cycle duration have decreased significantly. The decrease in capacity utilization should be considered as the most noticeable negative effect. It occurs as a result of a significant diminution of the charge which passes in the course of the charging stage of the battery. The causes of the diminution are various, but primarily it is due to a decrease in the total amount of Br atoms in the system. Therefore, we should turn to the analysis of the Br atoms’ amount in the catholyte and in the cell as a whole before and after the measurements (Table 2).

**Figure 8:**
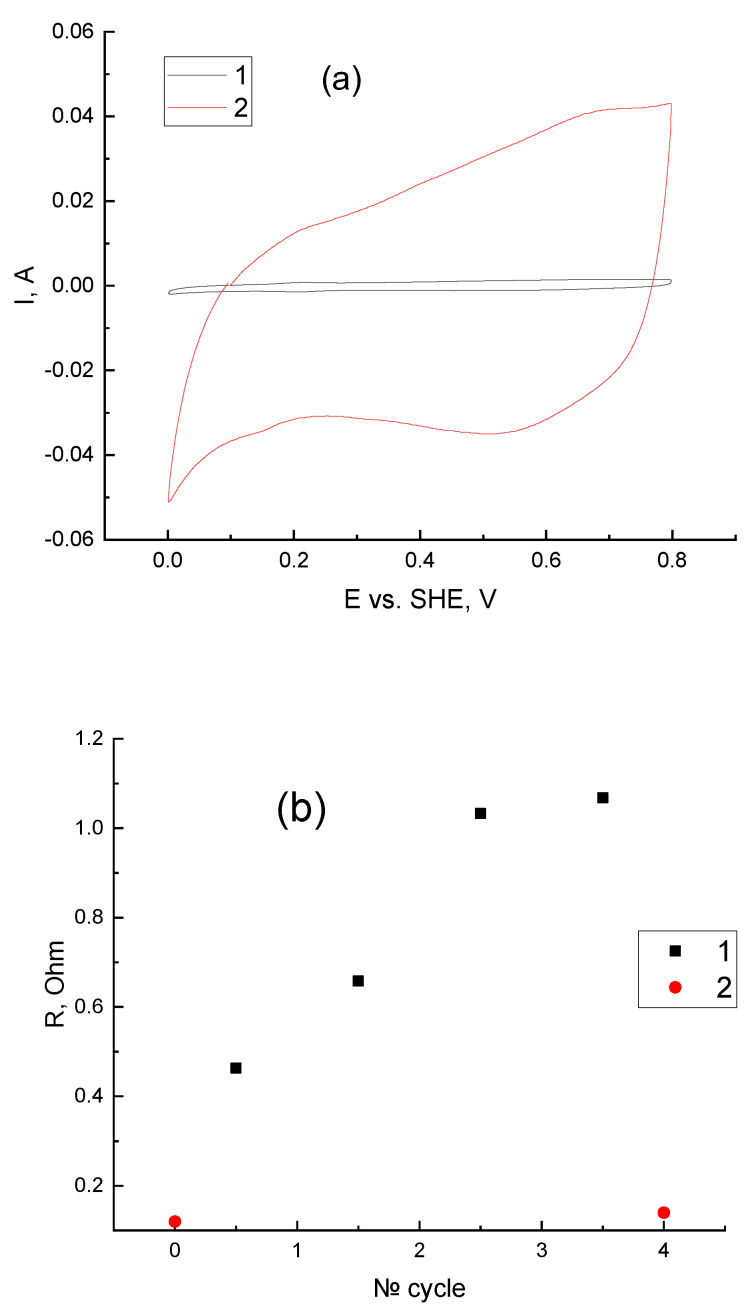
Comparison of characteristics of the H_2_-BrO_3_^−^ flow battery before and after cyclic charge—discharge tests: (**a**) cyclic voltammetry plots of H_2_-BrO_3_^−^—flow batteries before (1) and after (2) cycling, 3 M H_2_SO_4_ flux through the cathode, 0.5 L/h H_2_ flux through the anode, sweep rate 20 mV/s, 3 cycles. (**b**) Points 1 (black squares): total resistance of the cell calculated from the voltage jump at the moment when the current is switched between the charge and discharge regimes for cycles 1 to 4; Points 2 (red circles): cell resistance before and after 4 charge—discharge cycles measured by the impedance method.

The results of the analysis of the electrode potential variation during cycling are demonstrated in Figure 6a, curve 1. The charging segments of this dependence indicate that the overvoltage of the catholyte oxidation decreases from cycle to cycle. This decrease is caused by the growth of the surface of the positive electrode. This is clearly evidenced by an increase in its capacitive current of more than 20 times after cycling, compared to that before cycling, according to the CV measurements in Figure 8a.

Subsequently, the harging current density or the charge transfer voltage is reduced. It should be emphasized that the increasing capacitance/development of the surface are caused by the destructive effect of high potentials and strong oxidizers on carbon material. However, this destructive effect did not cause any noticeable increase in cell resistance during the four cycles performed (Figure 8b): the increase in its impedance during measurements at a high frequency did not exceed 16%. On the other hand, the total resistance of the cell when current was passed (determined by the ratio of the power surge during the transition from charge to discharge to the current surge) grew noticeably—almost three times. Considering the previously noted decrease in the cathode polarization and the relative constancy of the MEA ohmic resistance we assumed that the reason for the increase in total resistance was anode polarization, which increases from cycle to cycle. Similar problems were noted in [16,18] and are apparently caused by the catalytic surface poisoning of platinum with bromine compounds. The same effect can appear because of the anode catalyst “flooding” with water carried with the flow of hydrogen ions from the cathode to the anode while charging [69].

Thus, we emphasize two main reasons for the strong decrease in capacity utilization: the evaporation of bromine and the increase in polarization in the anode due to a decrease in the electrochemically active surface of the platinum catalyst due to the adsorption of bromine compounds, as well as the possible penetration of excessive water into the anode space transferred by the cation flow (H^+^) in the course of the charging stage.

## 4. Conclusions

For the first time, charge—discharge tests of a hydrogen-bromate flow battery have been carried out. Using porous flow-through carbon as the cathode material, it is possible to achieve a relatively high energy efficiency in the first few cycles. The second cycle is characterized by an energy efficiency of 51% and a capacity utilization of 70% at 75 mA/cm^2^. The reasons for the decrease in capacity utilization from cycle to cycle are bromine loss and anode poisoning. These negative effects can be minimized by the use of less bromine-absorbing materials and bromine-tolerant anode catalysts, respectively. To create a durable hydrogen-bromate flow battery, it is necessary to use electrodes [56,57,58,59,60,61] to improve the coulombic, voltaic and energy efficiency.

## Figures and Tables

**Table 1:** Characteristics of the charge—discharge cycle of a hydrogen-bromate flow battery according to Figure 6.

No.	Q_charge_, ×10^3^ Kл	Q_discharge,_ ×10^3^ C	U_charge_, V	U_discharge_, V	ηQ, %	ηU, %	ηE, %	Q_discharge_/Q_tot,_ %
1	3.1	1.9	1.6	1.2	61	75	46	90
2	2.3	1.6	1.6	1.1	70	69	48	70
3	1.1	0.9	1.6	1	82	62	51	40
4	0.5	0.4	1.5	1	80	67	54	20

**Table 2:** Estimation of losses of Br-containing compounds after charge-discharge tests.

	Amount of Bromine Atoms Before Cycling, mmol	Amount of Bromine Atoms After Cycling (Figure 5), mmol	Relative Change in Bromine Content, %
Catholyte	3.98	3.07	77
Escaping hydrogen trap	0	0.06	1.6
Anodic half-cell	0	0.06	1.6
Total amount of bromine atoms	3.98	3.19	80.2
